# The Blood Gene Expression Signature for Kawasaki Disease in Children Identified with Advanced Feature Selection Methods

**DOI:** 10.1155/2020/6062436

**Published:** 2020-06-28

**Authors:** Bing Hu, Yun Li, Guilian Wang, Yanqing Zhang

**Affiliations:** ^1^Department of Pediatrics, Yichun People's Hospital, Yichun 336000, China; ^2^Department of Obstetrics and Gynecology, Yichun People's Hospital, Yichun 336000, China

## Abstract

Kawasaki disease (KD) is an acute vasculitis, accompanied by coronary artery aneurysm, coronary artery dilatation, arrhythmia, and other serious cardiovascular diseases. So far, the etiology of KD is unclear; it is necessary to study the molecular mechanism and related factors of KD. In this study, we analyzed the expression profiles of 75 DB (identifying bacteria), 122 DV (identifying virus), 71 HC (healthy control), and 311 KD (Kawasaki disease) samples. 332 key genes related to KD and pathogen infections were identified using a combination of advanced feature selection methods: (1) Boruta, (2) Monte-Carlo Feature Selection (MCFS), and (3) Incremental Feature Selection (IFS). The number of signature genes was narrowed down step by step. Subsequently, their functions were revealed by KEGG and GO enrichment analyses. Our results provided clues of potential molecular mechanisms of KD and were helpful for KD detection and treatment.

## 1. Introduction

Kawasaki disease (KD) is an acute vasculitis, accompanied by coronary artery aneurysm, coronary artery dilatation, arrhythmia, and other serious cardiovascular diseases [1, 2]. It was first described by Japanese doctor Kawasaki in the late 1960s and has since been reported around the world with an increasing incidence [3, 4]. According to the recent survey, Japan owns the highest incidence of KD with 265 cases per 100,000 kids under the age of five [5]. KD initially manifested as high fever, cervical lymphadenopathy, and mucocutaneous inflammation [6]. Aspirin therapy and intravenous immunoglobulin (IVIG) injection play a key role in the effective treatment of KD, reducing the incidence of coronary artery complications from 5% to 25% [7]. KD occurs not only in infant and childhood period but even in adolescence. The young age of onset may suggest that susceptibility may be related to the maturity of the immune system [8].

So far, the etiology of KD is unclear, but epidemiological features indicate that there may be a connection between it and as-yet-undefined pathogen infections. In the surveys of Uehara and Belay, the incidence of KD reached a peak in winter and spring, which was similar to that of many respiratory diseases. This seasonal feature provides a new thought that KD may be caused by one or several pathogens related to respiratory diseases [2, 8, 9]. According to statistics, 8-42% of patients was associated with respiratory virus infection and 33% with bacterial infection [10–13]. Viral infection leads to abnormal lymphocyte subsets and inflammation, which were positively correlated with the occurrence of vascular inflammation in KD [14]. Rowley et al. found that the upregulation of expression of the interferon-stimulated gene was detected in acute lung tissue of KD, which illustrated the presence of cellular immune response after viral infection. They also observed that coronary artery inflammation of KD was characterized by antiviral immune response, including the upregulation of related genes induced by type I interferon and activation of cytotoxic T lymphocytes [15–17]. A related study suggested that some common respiratory viruses, such as enteroviruses, adenoviruses, coronaviruses, and rhinoviruses, were associated with KD cases [11]. It is reported that among these viruses, human coronavirus (HCoV)-229E may be involved in the occurrence of KD [18]. All of these strongly support the hypothesis that the infection of viruses and bacteria may be related to KD.

Up to date, there is no clinical specific diagnostic test for KD, and the diagnosis is still highly dependent on the symptoms and ultrasound imaging results [19]. Therefore, it is still necessary to study the molecular mechanism and related factors of KD. In this study, we analyzed the expression profiles of DB (identifying bacteria), DV (identifying virus), HC (healthy control), and KD (Kawasaki disease) samples. By comparing their expression difference, we obtained 332 key genes related to KD and pathogen infections. Subsequently, their functions were revealed by KEGG and GO enrichment analysis. Our study provides a direction for the study of potential molecular mechanism of KD occurrence.

## 2. Materials and Methods

### 2.1. Dataset

The gene expression profiles of 75 DB (identifying bacteria), 122 DV (identifying virus), 71 HC (healthy control), and 311 KD (Kawasaki disease) samples were downloaded from GEO (Gene Expression Omnibus) under the accession number GSE73464 [20].These samples were measured with two microarray platforms: Illumina HumanHT-12 V3.0 expression beadchip and Illumina HumanHT-12 V4.0 expression beadchip. Only the common 25,159 genes were analyzed. We performed quantile normalization to make sure the samples from a different batch were comparable using the R function “normalize.quantiles” in package preprocessCore (https://bioconductor.org/packages/preprocessCore/).

### 2.2. Boruta Feature Filtering

Since there were many genes and most of them were not associated with KD, we applied Boruta feature filtering [21] to detect all the relevant genes first. Boruta feature filtering is an advanced feature selection method wrapped with random forest. First, the real dataset was shuffled. Then, the importance of each feature was calculated. The features with real importance scores significantly higher than the shuffled ones were kept. Iteratively, all relevant features were selected. With Boruta feature filtering, we got a much smaller number of features for further analysis. We used python package Boruta (https://pypi.org/project/Boruta/) to apply the Boruta feature filtering.

### 2.3. Monte-Carlo Feature Selection

We adopted the Monte-Carlo Feature Selection (MCFS) [22] to rank the relevant features. It generated a number of randomly selected feature sets and then constructed many classification trees [23–25]. By ensembling these classification trees, the importance of each feature was calculated. In general, a feature was important if it had been selected by many classification trees. Suppose *d* was the total number of relevant features selected by Boruta, *m* features (*m* < <*d*) were randomly selected for *s* times, and *t* trees for each of the *s* subsets were constructed. Finally, there were *s*∙*t* classification trees. The relative importance (RI) of feature was

(1) $$\begin{array}{c} {\text{RI}}_{g}=\overset{st}{\underset{\tau =1}{\sum }}{\left(w\text{Acc}\right)}^{u}\underset{n_{\text{g}}\left(\tau \right)}{\sum }\text{I}\text{G}\left(n_{\text{g}}\left(\tau \right)\right){\left(\frac{\text{no}.\text{ in }n_{\text{g}}\left(\tau \right)}{\text{no}.\text{ in }\tau }\right)}^{v}, \end{array}$$

where *w*Acc was the weighted classification accuracy of decision tree *τ*, IG(*n*_g_(*τ*)) was the information gain of node *n*_g_(*τ*) which was a decision rule of feature *g*, (no.in *n*_g_(*τ*) was the number of samples under node *n*_g_(*τ*), (no.in *τ*)was the number of samples in decision tree *τ*, and *u* and *v* were adjusted parameters.

Based on RI, the features were ranked as *F*

(2) $$\begin{array}{c} F=\left[f_{1},f_{2}\cdots f_{N}\right], \end{array}$$

where *N* was the total number of relevant features, and the feature with smaller index had greater RI.

### 2.4. Incremental Feature Selection

After the features were ranked by MCFS, it was still difficult to decide how many features should be selected. To avoid arbitrary chosen cutoffs, we applied Incremental Feature Selection (IFS) [26–30]. For the selected and ranked feature list *F*, we created a series of feature subsets by iteratively adding top ranking features into the previous feature subsets and then evaluated their performance by building SVM classifiers and applying a leave-one-out cross validation (LOOCV). The feature subset with the highest LOOCV accuracy was selected.

## 3. Results and Discussion

### 3.1. The Irrelevant Genes of Kawasaki Disease Were Filtered by Boruta

The genome-wide expression measurements of genes provided a powerful way to understand the molecular functions of Kawasaki disease. But most of the genes were not associated with KD and were noise for sophisticated bioinformatics analysis. Therefore, we applied the Boruta algorithm to filter the irrelevant genes and kept the relevant genes. After performing Boruta, the dimension of genes was reduced to 1,485 from the original 25,159 genes.

### 3.2. The Genes Were Ranked Based on Their Importance in Kawasaki Disease

For the 1,485 KD relevant genes, we wanted to know how strong it was associated with KD. To rank them based on their importance, we used the MCFS method. It can rank the genes based on their contributions in a series of classification trees. Since it was an ensemble learning method, the results were reliable and robust. The ranked genes and their relative importance were listed in Table S1. The top 663 genes were marked as “top Ranking” genes by MCFS.

### 3.3. The Kawasaki Disease Signature Genes Selected with IFS Method

**Figure 1:**
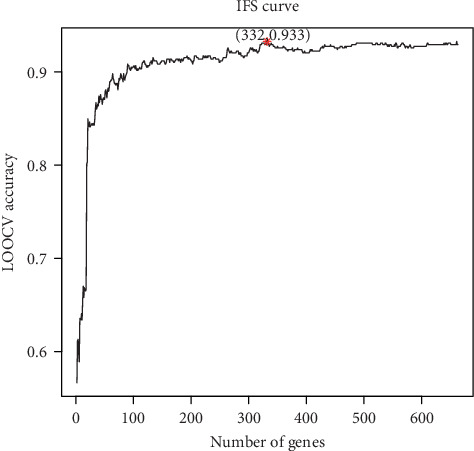
The IFS curve of Kawasaki disease signature gene selection. The *x* axis was the number of top genes and the *y* axis was the LOOCV accuracy. The highest LOOCV accuracy was 0.933 when 332 genes were used. Therefore, these 332 genes were selected as Kawasaki disease signature genes.

The number of genes, 663, was still too large for gene signature. To further reduce the number of genes, we applied IFS procedure on the top 663 genes in Table S1. We tried different numbers of top ranked genes and calculated their SVM LOOCV accuracy. The IFS curve was shown in Figure 1. The highest LOOCV accuracy was 0.933 when 332 genes were used. Therefore, these 332 genes were selected as the final Kawasaki disease signature genes.

The confusion matrix of the 332 genes is shown in Table 1. It can be seen that most samples were correctly classified. Among the four groups, only DB had a relatively poor performance. The other three groups all had excellent performance.

### 3.4. The Biological Significance of 332 Selected Genes

We found that some genes have been confirmed to be associated with KD. For example, Haptoglobin (HP), an acute-phase protein synthesized by the liver, responds to inflammatory cytokines and has been thought to be associated with vascular disease [31, 32]. Huang et al. made a comprehensive evaluation of the acute phase reactants in patients with KD. It was found that the level of serum HP in KD cases was significantly higher than that in other febrile diseases. The ratio of HP/apolipoprotein A-I could accurately distinguish KD from other febrile diseases and could be used as an auxiliary laboratory index in the acute phase of KD [33]. The early diagnosis and treatment of KD are very important for better prognosis and better survival rate in children. By studying the relationship between HP phenotype and coronary artery abnormal (CAA) formation in patients with KD, Lee et al. found that the clinical symptoms of HP-1 patients were delayed or incomplete, and the late diagnosis of KD was related to Haptoglobin phenotype [34]. BAX is an essential medium for endogenous apoptosis of the permeable mitochondrial outer membrane [35]. In the study of Tsujimoto et al., they measured the expression levels of antiapoptotic protein A1 and proapoptotic protein BAX and the ratio of A1/BAX in the viral infection group, bacterial infection group, KD group, and healthy children group. The results showed that the ratio of A1/BAX in patients with acute KD was significantly increased, suggesting that spontaneous apoptosis of PMN was inhibited in patients with acute KD [36].

To comprehensively study the biological functions of these 332 selected genes, we enrich them onto KEGG pathways and GO terms using a hypergeometric test. The enrichment results with FDR smaller than 0.05 are given in Table 2.

The results of KEGG enrichment analysis showed that the key genes were significantly correlated with influenza A, Epstein-Barr virus (EBV) infection, hepatitis C, systemic lupus erythematosus, and measles. Many previous studies have shown that KD is associated with influenza, coronavirus, and EBV. A case report in Wang et al. described a case of incomplete KD (IKD) consistent with influenza A (H1N1) pdm09 virus, suggesting that influenza infection may be a potential cause of KD [14]. In addition, a study from Korea shows that the monthly incidence of KD showed significant correlation with the monthly overall viral detection, including human bocavirus, enterovirus, and influenza [37]. A case-control study shows that specimens of respiratory secretions from 8 of 11 children with KD and from 1 of 22 control subjects tested positive for New Haven coronavirus (HCoV-NH). These data suggest that HCoV-NH infection is associated with KD [38]. Unfortunately, in another study in Taiwan, researchers did not find any association between HCoV-NH infection and KD [39]. A recent study reported an unusually high incidence of Kawasaki disease in children in a French centre for emerging infectious diseases: 17 cases in 11 days. In 82% of the cases, IgG antibodies for SARS-CoV-2 were detected, suggesting an association between the virus and this syndrome in children [40]. As for the correlation between KD and EBV, Huang et al. found that EBV sequences were detected in 83% repeatedly tested KD patients within 3 months after onset; the proportion is much higher than that of the control group [41]. These virological studies indicate that an unusual EBV-cell interaction may occur in KD. Besides, the prevalent ages at onset for KD and EBV infection are known to be similar in Korea and Japan [42]. Pavone et al. suggest in the case report that KD is caused by a new virus that may cross-react with EBV. Therefore, in febrile children with EBV infection or similar conditions to consider the possibility of Kawasaki disease, it is necessary to make a differential diagnosis in order to start intravenous immunoglobulin therapy in time [43]. Lee et al. reported that children with KD had normal immune response to EBV infection. Children with a history of KD appeared to be infected with EBV later than those with no history of KD [44]. It has been reported that in patients with a history of KD, the occurrence of the second autoimmune disease should also be considered. In addition, the initial manifestations of lupus may mimic KD [45].

Furthermore, through the enrichment analysis of GO pathway, it is found that these key genes were enriched in the functions related to cytoplasmic composition, immune response, enzyme activity, and molecular binding. There is a lot of evidence that the innate immune system plays an important role in initiating and mediating host inflammation. The acute phase of Kawasaki disease is characterized by inhibitory T cell deficiency. The obvious activation of T cells, B cells, and monocytes is related to the increase of cytokines secreted by these immune effector cells. This immune activation promotes the injury of vascular endothelial cells in Kawasaki disease. The light and electron microscopic studies on the antigens in the ciliated bronchial epithelium of acute KD by Rowley and Shulman showed that the KD-associated antigens were located in the cytoplasmic inclusions consistent with the aggregation of viral proteins and associated nucleic acids [46].

## 4. Conclusions

To sum up, we analyzed the gene expression profiles of KD samples and identified the blood gene signature of KD. The functional analysis of these KD signature genes suggested that the correlation between KD and pathogen infection, especially the new influenza virus H1N1, should attract more attention. In addition, the potential mechanism of KD mediated by virus infection is also worthy of further study, which may provide scientific basis and new insights for the pathogenesis of KD. Our study also provides a direction for the study of etiology of KD in the future.

## Figures and Tables

**Table 1:** The confusion matrix of the 332 genes.

	ActualDB	ActualDV	ActualHC	ActualKD
PredictedDB	57	4	1	2
PredictedDV	13	116	5	6
PredictedHC	2	0	65	1
PredictedKD	3	2	0	302
Sample size	75	122	71	311
Accuracy	0.760	0.951	0.915	0.971

**Table 2:** KEGG and GO enrichment results of the 332 genes.

Category	Function	FDR
KEGG	hsa05164 influenza A	0.012638
	hsa05169 Epstein-Barr virus infection	0.012638
	hsa05160 hepatitis C	0.012834
	hsa05322 systemic lupus erythematosus	0.012834
	hsa05162 measles	0.013912

GO BP∗	GO:0006955 immune response	7.29*E*-22
	GO:0002252 immune effector process	2.36*E*-21
	GO:0006952 defense response	1.09*E*-20
	GO:0002376 immune system process	1.82*E*-20
	GO:0045087 innate immune response	3.95*E*-18
	GO:0019221 cytokine-mediated signaling pathway	9.28*E*-17
	GO:0051707 response to other organisms	7.40*E*-16
	GO:0043207 response to external biotic stimulus	7.40*E*-16
	GO:0009607 response to biotic stimulus	3.25*E*-15
	GO:0060337 type I interferon signaling pathway	6.82*E*-14

GO MF	GO:0001730 2′-5′-oligoadenylate synthetase activity	0.008883
	GO:0003725 double-stranded RNA binding	0.008883
	GO:0050786 RAGE receptor binding	0.02261

GO CC∗	GO:0044433 cytoplasmic vesicle part	1.54*E*-08
	GO:0031982 vesicle	1.31*E*-07
	GO:0005737 cytoplasm	1.59*E*-07
	GO:0044444 cytoplasmic part	3.60*E*-07
	GO:0031410 cytoplasmic vesicle	1.81*E*-06
	GO:0097708 intracellular vesicle	1.81*E*-06
	GO:0042581 specific granule	2.16*E*-06
	GO:0030141 secretory granule	2.45*E*-06
	GO:0005829 cytosol	4.43*E*-06
	GO:0012505 endomembrane system	6.44*E*-06

∗Since there were too many enriched “GO BP” and “GO CC” terms, only the top 10 terms were listed.

## Data Availability

The gene expression data is available at https://www.ncbi.nlm.nih.gov/geo/query/acc.cgi?acc=GSE73464.

## Supplementary Materials

Supplementary Materials Table S1: the ranked genes and their relative importance calculated with MCFS.
