# Supplementary material for: The Blood Gene Expression Signature for Kawasaki Disease in Children Identified with Advanced Feature Selection Methods
Source: Biomed Res Int. 2020 Jun 28;2020:6062436. doi: 10.1155/2020/6062436 (PMC7327570; doi:10.1155/2020/6062436)
Supplement: Supplementary Materials — Table S1: the ranked genes and their relative importance calculated with MCFS. [file 6062436.f1.pdf]

Rank	Gene	Relative Importance
1	NAG18	0.36000308
2	C19orf31	0.3523193
3	TMSB4X	0.35129923
4	UBC	0.3473719
5	ORC6L	0.33218265
6	SHC1	0.33168957
7	IMAA	0.31012365
8	PDE4C	0.29936185
9	GGA1	0.2884386
10	LOC643509	0.28700924
11	ITIH5	0.28060415
12	SLC44A4	0.27057645
13	ARPC2	0.25027302
14	UNC45A	0.24592486
15	C21orf55	0.23184118
16	GNAS	0.22336727
17	AIRE	0.22249596
18	AKR1D1	0.22170645
19	IFI27	0.21943754
20	C21orf24	0.2133718
21	HP	0.20034078
22	PSMD12	0.1986508
23	STK24	0.1945225
24	LOC389517	0.1934089
25	CLUAP1	0.18326026
26	SLC25A3	0.17765063
27	LOC642947	0.17544626
28	CDAN1	0.1738169
29	GJC1	0.17201358
30	SHCBP1	0.16924067
31	CLTB	0.16489618
32	SERPINB6	0.16406159
33	GPX1	0.16340104
34	GPR84	0.15685973
35	GYG1	0.15558413
36	FTHL7	0.15443394
37	DCTN3	0.15443048
38	SLC16A12	0.15199228
39	SPOP	0.14969622
40	C3orf60	0.14728656
41	RNF34	0.14381029
42	PTPN6	0.14245895
43	C19orf59	0.1424459

44	SEC24C	0.1422294
45	LOC653086	0.14027531
46	MEF2D	0.14002062
47	BSG	0.13842101
48	REPIN1	0.13461603
49	MGC16703	0.13395126
50	C21orf7	0.13240428
51	OPLAH	0.1320285
52	SDHALP1	0.12590906
53	HPCAL1	0.12447115
54	TRMT5	0.123148166
55	RNF7	0.121042304
56	BAX	0.121017456
57	SETD3	0.12072239
58	BAT1	0.11960507
59	TSPO	0.119106606
60	ATP5G2	0.11906991
61	LOC348840	0.11737968
62	HK1	0.11501848
63	PDGFC	0.11370359
64	KCTD14	0.113638535
65	LY6E	0.11203363
66	OTOF	0.1119267
67	HIST2H2AC	0.111916855
68	UBE2I	0.11154278
69	IGLL1	0.111129664
70	DBP	0.11064012
71	ETFB	0.108941466
72	GAK	0.108040236
73	IFI44L	0.10718108
74	DACH1	0.107014544
75	NIPA2	0.10655466
76	RAB32	0.10434535
77	ARHGEF7	0.103304826
78	NOV	0.10299446
79	KCNH6	0.10264436
80	ADAM19	0.10218227
81	METTL7B	0.0988765
82	OAS1	0.09824749
83	SMPDL3A	0.09812359
84	SIGLEC1	0.097839795
85	AXL	0.096493274
86	C11orf82	0.09599785
87	CBX7	0.093002334

88	FBXO18	0.09274768
89	UBE1	0.091729194
90	CETP	0.0902334
91	RETN	0.09000583
92	TDRD9	0.08988873
93	ZGPAT	0.08756204
94	LAIR1	0.087546796
95	LOC440731	0.086878896
96	CD177	0.086783536
97	SNRPD2	0.086659566
98	RALGDS	0.08628216
99	ZNF683	0.086189166
100	CMTM2	0.08575817
101	BRD9	0.08574692
102	ZNF93	0.08178329
103	LOC388524	0.08017059
104	GAS7	0.07972901
105	S100A9	0.07755867
106	FKTN	0.07747077
107	ARG1	0.073877275
108	NRCAM	0.07322396
109	OAS2	0.072644256
110	TRPT1	0.07261585
111	ATL3	0.072215825
112	IGF2BP3	0.07206646
113	VPS16	0.071506806
114	ATP9A	0.07123237
115	KIAA1715	0.07103308
116	GOLGA7	0.07089891
117	TTN	0.06993392
118	ZNF682	0.06962261
119	IFI44	0.06910725
120	LAP3	0.06888484
121	FBXW2	0.06858472
122	EPSTI1	0.067888916
123	IRAK3	0.067868665
124	PQBP1	0.06613518
125	IFIT1	0.06607831
126	RPL8	0.065098025
127	CLEC5A	0.06485633
128	CDKN2AIPNL	0.06479295
129	C1QB	0.064703956
130	XAF1	0.06433807
131	SLC25A40	0.064074226

132	PPM1G	0.063343875
133	S100A4	0.062042013
134	MYL6	0.060847137
135	EIF6	0.06046572
136	FCGR1A	0.060414094
137	ANXA3	0.06001897
138	EIF3EIP	0.05935297
139	ANKRD22	0.058531307
140	RSAD2	0.05846795
141	SASH1	0.05812941
142	RAB36	0.058035623
143	SERPINB8	0.057630725
144	LOC26010	0.057589702
145	CMPK2	0.05750871
146	SLC4A5	0.05742742
147	CNOT1	0.057288013
148	GPR68	0.056929003
149	ALOX5	0.05672244
150	SLC2A3	0.056622308
151	PECR	0.05610668
152	IFI6	0.056087203
153	TMCO3	0.055846985
154	KREMEN1	0.05581532
155	ZDHHC19	0.055333257
156	EXOC7	0.055151504
157	SERPINB2	0.054429106
158	FCER1A	0.054373633
159	CCNDBP1	0.054334566
160	S100A12	0.05378089
161	IFIT3	0.053605434
162	SDHC	0.052901067
163	OMG	0.052420404
164	KIF3C	0.052370567
165	OAS3	0.05196094
166	PARP12	0.051566053
167	PARP9	0.05136812
168	LTA4H	0.051184792
169	GSTO1	0.05102667
170	HIST2H2AA3	0.050872713
171	TSPAN2	0.05064133
172	FLJ40142	0.05053511
173	PYGL	0.050156865
174	UBE2D3	0.049754173
175	IFIT2	0.049716905

176	PGS1	0.04952723
177	OSTalpha	0.049272887
178	HERC6	0.04918275
179	PAPSS1	0.0491819
180	IFIH1	0.04864303
181	DDX60	0.048078366
182	EIF4G1	0.048073087
183	SLC26A8	0.048071604
184	CS	0.048036903
185	CACNA1E	0.04798284
186	MX1	0.047832467
187	EIF2AK2	0.04756019
188	HSD3B7	0.04741451
189	PRDX5	0.047298238
190	EBI3	0.046638787
191	PHACTR4	0.046597432
192	DGUOK	0.046274602
193	IFITM3	0.045938693
194	P2RY14	0.04571856
195	SPCS2	0.04562912
196	FBXW11	0.04526848
197	IL8RBP	0.04501242
198	TSPAN32	0.04475396
199	CD44	0.044386595
200	IRF1	0.044127077
201	KIAA0101	0.043876085
202	NARF	0.04384761
203	ISG15	0.043664597
204	LMNB1	0.04334844
205	JUP	0.04292621
206	CACNG6	0.042668294
207	KIAA1160	0.04246677
208	SCARB2	0.042229626
209	TMEM16K	0.04210661
210	TCTN1	0.041925825
211	C14orf24	0.04183002
212	HERC5	0.04143305
213	IL1R2	0.041266758
214	MMD	0.041259322
215	ANAPC11	0.04124464
216	FYN	0.041107263
217	TLR7	0.040889826
218	CDC45L	0.040885188
219	IRF9	0.04081344

220	FCGR1B	0.040668793
221	LOC728744	0.040385537
222	CDK5RAP2	0.040112764
223	AGTPBP1	0.040101286
224	BPI	0.040086
225	PKM2	0.040026613
226	C5orf20	0.039875235
227	CERK	0.03957352
228	RCP9	0.039536376
229	DHRS7	0.039242722
230	ARID1A	0.039190896
231	KLHL22	0.03889985
232	SAPS2	0.0388262
233	SIGLEC9	0.038496323
234	CD163	0.038401812
235	HCFC1R1	0.03839096
236	ZDHHC16	0.038269605
237	SDPR	0.038172346
238	Septin.4	0.038076635
239	CBARA1	0.038003325
240	HIST1H2BD	0.037916653
241	NEXN	0.037851162
242	MMP8	0.037637953
243	VNN1	0.037182435
244	C1QC	0.03709862
245	CMTM1	0.037069988
246	MLSTD1	0.036816534
247	F2R	0.03665748
248	H2AFJ	0.03661465
249	FAM101B	0.03633061
250	PLSCR1	0.035979547
251	LOC728441	0.0357906
252	PLP2	0.035742246
253	SAMD9L	0.03563979
254	CCL8	0.035568275
255	ORAI2	0.03551344
256	LOC653559	0.035476644
257	MSH3	0.03540973
258	FAM20A	0.035292216
259	CA4	0.035242576
260	C20orf3	0.035205614
261	C14orf159	0.035145827
262	S100P	0.035079915
263	CCR3	0.035034046

264	UPP1	0.034975
265	GPR141	0.034788582
266	PSMB8	0.034709852
267	CD86	0.034673624
268	PRTN3	0.034537785
269	ITGB1	0.03428868
270	MPO	0.034207653
271	OIP5	0.034120988
272	RPS6KA5	0.034102175
273	HLA.DPA1	0.034094654
274	MGST1	0.034066692
275	FLJ22662	0.034025516
276	TLR5	0.033981156
277	RGL4	0.03383904
278	LACTB	0.033714093
279	MT1G	0.03371337
280	NSL1	0.033321723
281	BATF	0.033241685
282	TP53I3	0.033119198
283	PSMB9	0.033075266
284	CDCA5	0.032887366
285	TSC22D1	0.032678824
286	H1F0	0.03254214
287	C10orf33	0.032509536
288	HHEX	0.032507274
289	MGC10997	0.032076325
290	GALNT14	0.031892877
291	IER3	0.031679064
292	GLTSCR2	0.031581916
293	CCDC109A	0.031521752
294	DRAM	0.031505045
295	BLOC1S1	0.031479836
296	TCN2	0.03145601
297	PRSS33	0.03144762
298	PLA2G4B	0.03137265
299	SRPK1	0.031351607
300	MPZL2	0.031233622
301	THBS3	0.031097477
302	EMR4	0.031070545
303	PGRMC1	0.03103493
304	LYSMD2	0.030973768
305	PRAM1	0.030967968
306	LOC389816	0.03091637
307	CLC	0.030880367

308	IRF7	0.030707663
309	COL17A1	0.030538576
310	ZNF137	0.030521601
311	E2F2	0.03049022
312	HIST2H2BE	0.03045658
313	NLRP1	0.030358952
314	IL18R1	0.030356418
315	SLC31A1	0.030258885
316	PID1	0.030247077
317	IL18BP	0.03011915
318	SLC35A2	0.030115383
319	NCALD	0.029813858
320	C3AR1	0.029741887
321	GBP1	0.029734308
322	OASL	0.029708428
323	EMR2	0.029691834
324	SIGLEC10	0.029690385
325	LRPAP1	0.02967732
326	TGFBR2	0.029576197
327	EMR3	0.029517708
328	PSMD4	0.029485539
329	ADORA2A	0.029425591
330	CCPG1	0.029344184
331	NCR3	0.029272322
332	CACNA2D3	0.029246345
333	TOP2A	0.029242111
334	KCNG1	0.029209536
335	SEPHS2	0.029182533
336	RAC1	0.029155375
337	ZBTB4	0.029132035
338	OLFM4	0.02911899
339	BMX	0.029091803
340	PTAFR	0.029088318
341	STAT1	0.029047672
342	C5orf32	0.028895138
343	SIGLECP3	0.028879438
344	UPB1	0.028376482
345	WSB2	0.028249376
346	KIAA0232	0.0281506
347	GGTLC2	0.02812858
348	CECR1	0.02812028
349	UBE2L6	0.028068477
350	CCNA2	0.0280577
351	MS4A4A	0.02802227

352	C15orf52	0.027944464
353	SAT1	0.027867937
354	PSCD1	0.027861869
355	ZBTB40	0.027806828
356	LTB4DH	0.027765336
357	MOBKL2C	0.027673133
358	SPG7	0.027668742
359	MLLT6	0.02755398
360	DCTN2	0.027541725
361	ZBP1	0.027502295
362	GRB10	0.027416307
363	BAMBI	0.027353311
364	NDRG2	0.027271764
365	PACSIN1	0.027198061
366	BTN3A1	0.027136222
367	PRMT1	0.027122578
368	NSF	0.027054587
369	GINS2	0.02685083
370	STAMBP	0.026813498
371	LGALS3BP	0.026758075
372	LOH11CR2A	0.026719376
373	MXD4	0.026660439
374	LCN2	0.026637275
375	HSPBAP1	0.0265069
376	LOC728888	0.026506526
377	AMPH	0.026485957
378	NUSAP1	0.026440397
379	QRICH1	0.026361642
380	LGALS1	0.026347285
381	SLC26A11	0.026259165
382	ROGDI	0.026216175
383	SLC28A3	0.026120897
384	FCRL3	0.026109297
385	MAFB	0.025990954
386	GNG11	0.025919583
387	FLJ14213	0.025898416
388	KCNE1	0.02579158
389	UHRF1	0.025766678
390	P2RY8	0.025692089
391	DHX58	0.025647523
392	PLA2G7	0.025603427
393	ULK1	0.025590014
394	CTSD	0.025573341
395	SMARCD3	0.025505623

396	SELM	0.025504442
397	TYSND1	0.025468757
398	RAB37	0.025436476
399	LSM12	0.025390042
400	TPM4	0.025360866
401	GPBAR1	0.02526577
402	CUL1	0.025257826
403	HESX1	0.025162661
404	HSH2D	0.02511207
405	PLAC8	0.025100749
406	NDFIP2	0.0250864
407	EDNRB	0.025080856
408	CDC25B	0.025075257
409	VNN2	0.024867853
410	IL10RA	0.024832418
411	DPEP2	0.024824485
412	SIRPA	0.0247768
413	SQRDL	0.02476799
414	NLRC4	0.02475926
415	MGC29891	0.024746414
416	OGDH	0.024651393
417	CD63	0.024599146
418	MAN2B1	0.024592722
419	FGD2	0.024556074
420	PHF15	0.024533138
421	ADORA2B	0.024493862
422	FAM89A	0.024468986
423	LDHA	0.024371512
424	PDE4B	0.02436791
425	LOC130074	0.024326416
426	BAG5	0.02430322
427	RTN1	0.024294546
428	HDC	0.024256615
429	CLDN23	0.024244353
430	SP110	0.02421337
431	BACE2	0.024177311
432	IFI35	0.024171788
433	ACRBP	0.024105852
434	CKAP2L	0.024044825
435	CREB5	0.02399869
436	UBE2C	0.023960182
437	RAE1	0.023900956
438	CEACAM6	0.02387171
439	OLIG2	0.023705333

440	C9orf103	0.023701884
441	LAMP3	0.023656545
442	PTP4A3	0.023652451
443	EMR1	0.023595864
444	STAT2	0.02358888
445	GIMAP8	0.0235688
446	EEF1G	0.023494313
447	RUNX1	0.023392284
448	LOC648984	0.023344677
449	SULF2	0.023344545
450	CCNY	0.023247374
451	NQO2	0.023225736
452	TNK2	0.023212634
453	ADHFE1	0.023201043
454	LST1	0.023191262
455	GPSM2	0.023146255
456	MGAM	0.02313107
457	TYMS	0.023127886
458	METTL9	0.023119207
459	EDG4	0.02309999
460	RBBP6	0.023069656
461	FOXO3	0.022980511
462	ALOX15	0.022891793
463	SYT11	0.022874562
464	SH2D1B	0.022748875
465	CDK9	0.022735093
466	RASSF2	0.022714538
467	BCKDHA	0.022696938
468	C10orf10	0.02266069
469	CYB5R1	0.02260699
470	MAPK1	0.022584261
471	RIN2	0.022571808
472	RARA	0.022565318
473	MTHFD2	0.022561872
474	IFITM1	0.022504993
475	ADM	0.022462646
476	RHOC	0.022443818
477	CCDC56	0.022439562
478	LAPTM4B	0.02239458
479	DBNDD1	0.022388348
480	DEFA1	0.022380149
481	PHF1	0.022316735
482	ZNF185	0.022307046
483	CST7	0.02229904

484	MMP9	0.02222009
485	NAPRT1	0.022210669
486	HIST1H2BK	0.022202134
487	C20orf160	0.022178745
488	GAS6	0.022155203
489	HBXIP	0.022142716
490	C2orf18	0.022016643
491	KIAA0241	0.022001315
492	S100A8	0.021984177
493	IFNAR2	0.021941833
494	FOLR3	0.021935163
495	ELA2	0.021843072
496	LOC388969	0.021834154
497	DERA	0.021804798
498	CCL23	0.021798918
499	CYB5D2	0.021784848
500	GLS	0.021780184
501	TRAF1	0.02173578
502	MT1A	0.021666951
503	MED24	0.021650983
504	RNASE2	0.021634838
505	ALDH1A1	0.021608798
506	BMP6	0.021588076
507	ODC1	0.021530231
508	PLOD2	0.02151555
509	PLEKHA2	0.021472314
510	CDKN1A	0.021460438
511	DEF8	0.021458587
512	PNPLA1	0.0214518
513	PGLYRP1	0.021417955
514	SELP	0.021295
515	FEN1	0.021187518
516	SLPI	0.021176096
517	ARIH2	0.021153342
518	HK3	0.021114334
519	HIST1H4H	0.021113811
520	HPSE	0.021099743
521	EOMES	0.02105936
522	TRIM56	0.021029089
523	DEFA3	0.020974876
524	DEFA4	0.020938734
525	TAP1	0.020932104
526	ASH2L	0.02081357
527	PHCA	0.020741675

528	CD160	0.020733064
529	IMPA2	0.020730449
530	MMRN1	0.020722533
531	CCL5	0.020702122
532	MS4A3	0.020700378
533	KIAA1602	0.020682925
534	ADPRHL2	0.020677224
535	ACSL5	0.020669052
536	LFNG	0.020588497
537	C2	0.020553833
538	PGK1	0.020504497
539	SCRG1	0.02050159
540	LOC729776	0.02049552
541	SERPINB1	0.020386754
542	TAP2	0.020355921
543	CTSG	0.02031441
544	SERPING1	0.02026096
545	PPP2R4	0.02022494
546	PHTF1	0.020207427
547	TREML1	0.020172521
548	MT2A	0.020142818
549	ARL4A	0.020139668
550	NOMO2	0.02012099
551	CD2	0.020113902
552	DHCR7	0.02008388
553	MCM10	0.020073675
554	KLF4	0.020064138
555	EIF2C1	0.020028686
556	DDAH2	0.020000152
557	CST3	0.01998688
558	MELK	0.019966882
559	ALDH4A1	0.01996395
560	TRAPPC2	0.01996133
561	ZBTB48	0.019936897
562	SGSM2	0.01991187
563	YTHDC1	0.019891279
564	CLIP1	0.01984664
565	CLEC1B	0.019837521
566	IL1B	0.019825848
567	FLJ11286	0.01978219
568	P2RX1	0.019768542
569	SCO2	0.019747376
570	SPPL2A	0.019740261
571	PIM3	0.019618174

572	IFITM4P	0.019576231
573	GPR44	0.019568408
574	C14orf85	0.019534012
575	ROPN1L	0.01952392
576	SCAP	0.019508915
577	SLC22A4	0.019502837
578	C16orf7	0.019487511
579	GZMB	0.019479705
580	CNNM3	0.0194427
581	RTP4	0.019412305
582	MAFG	0.019377388
583	NAMPT	0.019325823
584	HIST2H2AB	0.019199995
585	HIST1H1C	0.019166892
586	QARS	0.019152198
587	NGFRAP1	0.019127889
588	CAPN13	0.019101094
589	LOC649181	0.019101024
590	POLE2	0.019089857
591	STXBP5	0.0190679
592	BCL6	0.01905768
593	PDE5A	0.01905051
594	BTN3A3	0.019035952
595	FBL	0.019019676
596	B3GNT8	0.019009892
597	CRY2	0.018989258
598	HES4	0.018964248
599	ZDHHC14	0.018951733
600	LOC649150	0.018929424
601	MYBPH	0.018908191
602	PLEKHG2	0.018905351
603	CEACAM8	0.018903904
604	RUNDC3A	0.018867668
605	AP3S2	0.01886132
606	ARS2	0.018845746
607	C9orf91	0.018836847
608	LOC728358	0.018835666
609	TFPI	0.018826332
610	CLCF1	0.018801413
611	CHEK1	0.018755874
612	IDH1	0.01863098
613	STOM	0.01862457
614	SCD	0.018578324
615	LYPLA1	0.01857456

616	MTE	0.01855141
617	NAT8B	0.01851833
618	ZAP70	0.018467085
619	TAGAP	0.01833723
620	ZMYND15	0.018289333
621	IL5RA	0.018278997
622	GIMAP4	0.018277934
623	LY9	0.018269109
624	TIMM10	0.018242996
625	EMILIN2	0.018193543
626	TMC6	0.018154025
627	SH3GLB1	0.018151646
628	C11orf51	0.01812179
629	CEP55	0.01807912
630	BST2	0.018066028
631	PERLD1	0.01806296
632	IFIT5	0.018059954
633	LOC642103	0.018047793
634	INTS7	0.01802978
635	TUBB1	0.017972874
636	INADL	0.01795889
637	PSTPIP2	0.017900676
638	SH3BGRL2	0.017899763
639	LDLR	0.017882066
640	KIAA0141	0.017877001
641	ACAA1	0.017873952
642	ERP27	0.017826132
643	LEPREL1	0.017792303
644	EIF4B	0.017783122
645	SNX3	0.017781984
646	Septin.5	0.017746191
647	PRG2	0.017718531
648	PRIC285	0.017662767
649	GBA	0.017649973
650	PPM1F	0.017630858
651	MT1E	0.017608412
652	C9orf156	0.017603733
653	ATP6V1C1	0.01758758
654	SFRS17A	0.017558632
655	PDGFD	0.017546464
656	SULT1B1	0.017529396
657	GAPDH	0.017503431
658	ACSL1	0.017473545
659	CAV2	0.017469993

660	KIF20A	0.017467868
661	NR3C2	0.017434055
662	CDC20	0.01739643
663	LOC116236	0.017383153
664	SLC4A2	0.017347686
665	MTL5	0.01732682
666	PLTP	0.017324345
667	AKT1	0.017323947
668	DAAM2	0.017323153
669	CXCL16	0.017286004
670	KIF2C	0.017280523
671	SPOCK2	0.017230138
672	GGT1	0.0171889
673	JAM3	0.01717332
674	DNASE2	0.017168844
675	TXNDC17	0.017159684
676	GTF3C6	0.017155942
677	CD247	0.017146787
678	S100A6	0.017141357
679	BIRC5	0.017125526
680	FLJ20674	0.017123537
681	VSIG4	0.017087653
682	STAG3L2	0.01708457
683	PLD3	0.01708175
684	CCL2	0.017079432
685	NR4A2	0.017064031
686	PAFAH2	0.017062293
687	ZDHHC3	0.017031075
688	OGFOD1	0.01702207
689	CDT1	0.016963784
690	RNASE3	0.016953526
691	ADIPOR2	0.016928375
692	ZNF313	0.016919544
693	IL11RA	0.016889645
694	UBTD1	0.016887113
695	RNF165	0.016872978
696	BEX1	0.016867967
697	FHL1	0.016866958
698	PGM2	0.01686642
699	DDEF2	0.01685734
700	SCPEP1	0.016842648
701	LOC642197	0.016828502
702	HVCN1	0.01682545
703	KPNB1	0.016797818

704	YPEL1	0.016767697
705	GPER	0.016767303
706	KCNH3	0.01674566
707	DRAP1	0.016673986
708	SAMD4A	0.016666135
709	RNF216	0.016609855
710	SLC3A2	0.016527282
711	LOC653600	0.016507644
712	FBXO6	0.016478736
713	PTRF	0.016466796
714	LOC645899	0.016463637
715	PPAP2C	0.016461385
716	WIPI1	0.01645992
717	CAMP	0.01642723
718	RNASE1	0.016408643
719	CASS4	0.016377172
720	PPM1M	0.016350962
721	SLC16A6	0.016342187
722	BST1	0.016339464
723	TARP	0.01629807
724	FCER1G	0.016258731
725	AGTRAP	0.016233604
726	SKP1A	0.01623047
727	ASCC1	0.016165517
728	IDH3G	0.016153228
729	PPA2	0.016136646
730	CTSL1	0.01610003
731	ZDHHC12	0.016055023
732	CSAD	0.016036918
733	LHFPL2	0.016030775
734	EXOSC4	0.016030578
735	ITM2B	0.016028922
736	DNMT1	0.016027289
737	PTGDS	0.015985394
738	RGL1	0.015955355
739	HNRPM	0.01595461
740	TMEM120A	0.015953658
741	C1orf122	0.015947158
742	LOC731049	0.015923742
743	CEACAM1	0.015922928
744	PCOLCE2	0.015881274
745	TCN1	0.015874537
746	PSCDBP	0.015859433
747	TMEM43	0.015841225

748	CD82	0.015836744
749	ID3	0.015831484
750	SGSH	0.015827818
751	PRC1	0.015817499
752	SYN1	0.015803896
753	ASB1	0.015792836
754	C1orf176	0.015757743
755	MCM4	0.015746105
756	RNF149	0.015734931
757	TRIM22	0.0157217
758	B3GALTL	0.01571544
759	LOC441019	0.015693847
760	MATK	0.015672201
761	TRIM26	0.01560649
762	IL1RAP	0.015592199
763	SLC25A20	0.015589159
764	HIST1H3H	0.015582838
765	BCAT1	0.015559865
766	NTN2L	0.015555343
767	KLHL2	0.015553681
768	DCUN1D1	0.015513774
769	CYP1B1	0.015507618
770	HCG27	0.015501901
771	MCTP1	0.015493603
772	SNORD16	0.015432087
773	PHF11	0.015429528
774	STX16	0.01542332
775	AZU1	0.015420154
776	ZMYM6	0.015401263
777	PIK4CA	0.015395064
778	EFTUD2	0.015388976
779	MYL6B	0.015375888
780	NUMB	0.015368279
781	PCCB	0.01535732
782	TXNDC4	0.015304701
783	ZNF650	0.015264327
784	CDCA7	0.015237022
785	ANKRD33	0.015230916
786	DIRC2	0.015225514
787	LOC728226	0.015215064
788	PRKCZ	0.015184731
789	NCAPG	0.015095498
790	TKTL1	0.015091131
791	D4S234E	0.015071414

792	NASP	0.015060757
793	MAD2L2	0.015048878
794	LBH	0.0150397
795	EPN2	0.015033911
796	LOC728519	0.015031331
797	SDHD	0.014987564
798	PRKCH	0.014972194
799	MAPK8IP3	0.014963495
800	DNAJB2	0.01495572
801	MAOA	0.014932171
802	HNRNPL	0.014926896
803	MX2	0.014918705
804	THOC6	0.014916116
805	PSME1	0.014914813
806	GOLGA8A	0.014914719
807	LILRB2	0.014887289
808	PPBP	0.014829229
809	HIST2H3C	0.014820373
810	LOC284023	0.014759242
811	ABCB1	0.01475171
812	NFKBIZ	0.0147312
813	LOC651309	0.014725514
814	RASSF1	0.014724929
815	C6orf173	0.014713562
816	SPAG7	0.014681242
817	PDZD4	0.014673704
818	RUNX3	0.014671263
819	LOC644615	0.014669672
820	P2RY10	0.014654827
821	HIPK3	0.014635556
822	SLC40A1	0.014607379
823	NPEPPS	0.014570701
824	LOC653610	0.014561166
825	ZNF324	0.014556006
826	TNFAIP6	0.014539249
827	HABP4	0.014538573
828	PPP2R2D	0.014533855
829	LAPTM5	0.014529815
830	BRD7	0.014519431
831	WDR51A	0.014494903
832	ASB8	0.014485191
833	RGL2	0.014482684
834	TBC1D7	0.014459589
835	GPAA1	0.014411835

836	CD74	0.014402886
837	CATSPER2	0.014402645
838	IPO11	0.014398616
839	MCRS1	0.014393479
840	GZMK	0.014369302
841	POFUT1	0.014366225
842	ACACB	0.014357067
843	GIMAP6	0.014335445
844	C9orf127	0.014334979
845	RBM39	0.014329486
846	MAPK14	0.014297327
847	FCRL6	0.014296066
848	TXNDC12	0.014248626
849	GZMH	0.014241454
850	CCNB2	0.014231688
851	M6PRBP1	0.014218399
852	GPR42	0.014208751
853	ITGAX	0.014201901
854	SLC30A1	0.014196856
855	SPRYD5	0.014162273
856	HYAL2	0.014142439
857	TRIP13	0.014095361
858	GIMAP5	0.014092067
859	LOC653874	0.014032519
860	NAIP	0.014015415
861	SAMSN1	0.014013672
862	DYM	0.013972525
863	COL4A3BP	0.013943324
864	PIWIL4	0.013920275
865	CDC25A	0.013877048
866	DKFZp586I1420	0.013860132
867	CC2D2B	0.013848544
868	APH1B	0.01384165
869	GADD45A	0.013834119
870	NCOA7	0.013820385
871	LOC400455	0.013814438
872	CDH6	0.013814194
873	DSC2	0.013811191
874	MFNG	0.013802412
875	ST3GAL5	0.013802212
876	ZCCHC2	0.013793991
877	KIAA0556	0.013789807
878	C16orf75	0.01377513
879	MPZL1	0.013745721

880	C7orf54	0.01373986
881	HCG4	0.013726248
882	PNPLA6	0.013723044
883	EIF3K	0.013722493
884	OLR1	0.013722316
885	PFKFB2	0.013703841
886	FLOT1	0.013702858
887	RNU12	0.013683008
888	IGFBP7	0.013650243
889	COBRA1	0.013643943
890	IFI16	0.013633167
891	DNAH1	0.013615419
892	NUP205	0.013612662
893	CLTA	0.013611604
894	TUBGCP6	0.013601543
895	CALD1	0.013592073
896	LOC731486	0.013583198
897	LOC650029	0.013571529
898	KLRG1	0.01354102
899	FKBP9L	0.013505356
900	SLC36A4	0.013483145
901	FAM100A	0.013458293
902	PEX16	0.013446705
903	IL4R	0.013441504
904	GGH	0.013440516
905	FBXW5	0.013408241
906	EPDR1	0.013401316
907	C3orf19	0.013398958
908	AGPAT2	0.013390445
909	CTRC	0.013380507
910	ORM2	0.013376521
911	NCK2	0.013368621
912	PPP1R13B	0.013349657
913	AMPD3	0.01333878
914	STIM2	0.013324203
915	ACSS2	0.013322527
916	HIST1H2BC	0.013316649
917	TIFA	0.013290409
918	CMAS	0.013284997
919	NUBP1	0.013275925
920	FCGBP	0.013247535
921	LOC400948	0.013213026
922	ATP2C1	0.013199727
923	ELF1	0.013195209

924	DDX58	0.013177802
925	PAQR4	0.013170168
926	PSMB7	0.013138246
927	C22orf28	0.013089403
928	LMTK3	0.01307773
929	ALPL	0.01302652
930	ACPP	0.013015856
931	TSEN54	0.012998685
932	GLA	0.012997121
933	LTF	0.012985386
934	AMICA1	0.012980652
935	OTUD1	0.012972485
936	NOMO1	0.012953504
937	PARP14	0.012949769
938	NSUN7	0.012943476
939	LRRC26	0.012943079
940	KIF15	0.012938856
941	KCNJ15	0.012934249
942	TP53I13	0.012911253
943	ATP5O	0.012910927
944	EDG8	0.012910841
945	DPM3	0.012908385
946	FCGR3A	0.012867292
947	M160	0.012846922
948	APOL1	0.012841008
949	PTTG3	0.012838488
950	NUB1	0.012828819
951	TBL1X	0.012796148
952	ASPM	0.012794794
953	DPEP3	0.012789182
954	COX15	0.012786388
955	ANXA5	0.012785632
956	GRWD1	0.012779757
957	CTSK	0.012758706
958	TMEM150	0.012748265
959	BLCAP	0.012735387
960	SARM1	0.012693447
961	NBPF10	0.012682198
962	CD96	0.012676305
963	MKL1	0.012662598
964	SYTL2	0.012648382
965	ITGB5	0.012632972
966	LOC387820	0.012620901
967	GBP4	0.012611347

968	LOC90586	0.012607112
969	DDX24	0.012604932
970	PARP10	0.012592932
971	ENTPD7	0.012587684
972	C21orf2	0.012574775
973	LOC440503	0.012572026
974	SAMD9	0.012555404
975	CXorf12	0.012551255
976	LOC400759	0.012549098
977	EEF1D	0.012544346
978	PAQR8	0.012517408
979	TNFRSF21	0.012480469
980	POMP	0.012478161
981	SPARC	0.012452526
982	LOC654103	0.012448387
983	GPI	0.012444296
984	C5orf30	0.012443111
985	FAM39DP	0.012438344
986	TOR1B	0.012417256
987	MAPBPIP	0.012372838
988	USP32	0.012328344
989	GSDMDC1	0.012311959
990	LRRC42	0.012290056
991	CEBPE	0.012281947
992	ALAS2	0.012227359
993	EFCBP1	0.012219781
994	EGLN2	0.012209061
995	TNFSF4	0.012207162
996	CYP2J2	0.01220471
997	CD6	0.012191396
998	LOC644774	0.012172819
999	GRN	0.012156684
1000	TCF20	0.012148983
1001	SETD8	0.012146484
1002	GSDML	0.012120246
1003	GYS1	0.012105678
1004	F13A1	0.012082905
1005	FAM102A	0.012081907
1006	LOC653316	0.012042712
1007	SFRS5	0.01203652
1008	TGFBR3	0.011995534
1009	SOD2	0.011978569
1010	ISG20	0.011968982
1011	KIAA1958	0.011963286

1012	IL2RB	0.01195496
1013	TK1	0.011950892
1014	CLEC4D	0.011946553
1015	HIST1H2BE	0.011927377
1016	EZH2	0.011896962
1017	TBPL1	0.011892813
1018	TNRC6A	0.011886051
1019	UBE4B	0.011873256
1020	SLC38A10	0.011871461
1021	PTGDR	0.011861367
1022	NDUFA1	0.011860793
1023	NDUFS4	0.011839767
1024	MOV10	0.01177349
1025	CASP5	0.011758487
1026	PITPNC1	0.011756332
1027	MGC3020	0.011747954
1028	TMEM140	0.011681878
1029	RCC2	0.011676923
1030	NAP5	0.011675384
1031	NEDD8	0.01164528
1032	MMP25	0.011588017
1033	SP140	0.011586698
1034	MAGT1	0.011573812
1035	CTGLF3	0.011557052
1036	ERAL1	0.011532807
1037	CXCL10	0.011523033
1038	CDCA3	0.011519279
1039	MPST	0.011512375
1040	TPM2	0.011491964
1041	ACP6	0.011455786
1042	TNPO1	0.011422577
1043	MLKL	0.01133194
1044	DUSP3	0.011330252
1045	FAM43A	0.011329893
1046	SLC25A42	0.011311555
1047	TUBG1	0.011310267
1048	CHN2	0.011293788
1049	CKAP4	0.011289164
1050	LOC285176	0.011281674
1051	GBP5	0.011241755
1052	MKNK1	0.011234328
1053	FFAR3	0.011230953
1054	N4BP2	0.011220849
1055	LMO2	0.011191943

1056	EXOC6	0.011174746
1057	CENPM	0.011171455
1058	STIL	0.011164754
1059	CMTM4	0.011149094
1060	RBM10	0.011144616
1061	RPL3	0.01111458
1062	ADAR	0.011092219
1063	KLF12	0.011085765
1064	MAP3K4	0.011066765
1065	MCM6	0.01105802
1066	LOC642684	0.011057086
1067	AURKA	0.01104202
1068	SLFN5	0.011040498
1069	HJURP	0.011012559
1070	IL10RB	0.010999957
1071	DSE	0.010998072
1072	LOC23117	0.010986466
1073	POLB	0.010980488
1074	CNDP2	0.010979126
1075	BCAS4	0.010973783
1076	KIAA1530	0.010968846
1077	KIFC1	0.010959549
1078	SIL1	0.010954495
1079	MLL	0.010948492
1080	IAH1	0.010946958
1081	ZNF232	0.010946235
1082	TPP1	0.010933366
1083	FBXL5	0.010928297
1084	RALGPS1	0.010905361
1085	ETV7	0.010884118
1086	C16orf72	0.010844552
1087	IDH3A	0.010832509
1088	TRIM5	0.010829678
1089	NEDD4	0.010827194
1090	GUCY1A3	0.010826338
1091	B4GALT5	0.010802129
1092	LRRFIP2	0.010797371
1093	TNFAIP1	0.010784218
1094	PYHIN1	0.010776212
1095	HIST1H3D	0.010775675
1096	CICE	0.010751937
1097	ARRDC4	0.010734148
1098	MXD3	0.010728274
1099	TXN	0.010718829

1100	PIK3R1	0.010700767
1101	PLEKHA1	0.010687778
1102	PUM1	0.010671654
1103	PTPRA	0.010671137
1104	ACVR1B	0.01066601
1105	CCR6	0.010655877
1106	COQ10A	0.010650252
1107	LOC643596	0.010646355
1108	PGM1	0.010631808
1109	SPG3A	0.010610228
1110	ZNF438	0.010565481
1111	GLB1L	0.0105542
1112	PEA15	0.010548866
1113	RFTN1	0.010547032
1114	PSME2	0.010528795
1115	XPNPEP3	0.01052742
1116	TMEM17	0.010519658
1117	KIFAP3	0.010517431
1118	ICA1	0.010514568
1119	SBK1	0.010514468
1120	EFEMP2	0.0105104
1121	TCTEX1D1	0.01048875
1122	DTL	0.010484005
1123	PRKACA	0.010480485
1124	NPIP	0.010476457
1125	PABPC4	0.010468722
1126	CD59	0.010462991
1127	GCS1	0.010428862
1128	PDK3	0.010421142
1129	HMGB2	0.010410629
1130	GPR63	0.010399694
1131	NELL2	0.010386252
1132	SLC11A1	0.010373138
1133	LOC347376	0.01037035
1134	RPL32	0.010332351
1135	TIAF1	0.010308945
1136	LTB4R	0.010300804
1137	RTN3	0.010292886
1138	ST6GALNAC3	0.010284602
1139	PLEKHA9	0.010284564
1140	HMMR	0.01024456
1141	GSN	0.010233641
1142	LOC648733	0.01022899
1143	KIF11	0.010228545

1144	KIF14	0.010227707
1145	EIF3F	0.010216726
1146	C12orf65	0.010198038
1147	HMGA1	0.010178953
1148	NDUFS5	0.010172631
1149	TRIP6	0.010133943
1150	ENY2	0.010125286
1151	PSD4	0.010114933
1152	PICALM	0.010099857
1153	LILRA5	0.010077616
1154	C20orf20	0.010063808
1155	NUFIP2	0.01005537
1156	MSRB3	0.010047778
1157	ATG16L1	0.010044058
1158	SFRS7	0.010039318
1159	IREB2	0.010035864
1160	CPSF2	0.010020252
1161	TMEM32	0.010011863
1162	GPR114	0.00999616
1163	RAB27A	0.009993166
1164	NPC1	0.009992271
1165	ATXN1	0.009963345
1166	DHX33	0.00994299
1167	APOL6	0.009919581
1168	RABGEF1	0.009908965
1169	CYBRD1	0.00989575
1170	YY1AP1	0.009894546
1171	CDK5RAP3	0.00988984
1172	WDR57	0.009878377
1173	KIAA0182	0.009860393
1174	BICD2	0.009850672
1175	PPM1H	0.009824904
1176	C11orf2	0.009810955
1177	UXT	0.009791121
1178	LILRA6	0.009760801
1179	HIST1H2AM	0.009749029
1180	KIAA0355	0.009734105
1181	PSMD5	0.009726184
1182	CYFIP2	0.009702235
1183	C1orf63	0.009696186
1184	CSNK1E	0.009620887
1185	GPR109A	0.009596605
1186	LIN7A	0.009569323
1187	LGALS9	0.009557037

1188	NLRP12	0.009552894
1189	LOC728734	0.009523504
1190	PCNXL2	0.009521368
1191	RFWD2	0.009509555
1192	AFF4	0.009501607
1193	NIPA1	0.00949253
1194	GTF2IRD2B	0.009446656
1195	BAZ1B	0.009429066
1196	ZNF684	0.00941415
1197	DOK2	0.00941283
1198	THYN1	0.009372008
1199	C18orf56	0.009367305
1200	DLG7	0.009345059
1201	NDUFA9	0.009333497
1202	WBSCR22	0.009288691
1203	TNFAIP8L3	0.00926352
1204	DUSP5	0.009262597
1205	LRRC37B	0.009246489
1206	MGC4677	0.009243102
1207	GALM	0.009218612
1208	DNAJC3	0.009201949
1209	C14orf156	0.009191473
1210	TPX2	0.009167875
1211	UBAC2	0.009141255
1212	SNORD35B	0.009133495
1213	RLF	0.009123242
1214	F5	0.009118201
1215	UQCRC1	0.009097804
1216	PCSK7	0.009095251
1217	AMZ2	0.009091831
1218	CASP4	0.009075471
1219	LOC441124	0.009038731
1220	PRCP	0.009020548
1221	SMG7	0.008993492
1222	HIST1H3F	0.008983509
1223	CDK5RAP1	0.00896439
1224	TREML3	0.008937012
1225	ARPC5	0.008896569
1226	ARL6IP5	0.008878482
1227	LMBRD1	0.008875868
1228	GCH1	0.008871401
1229	NECAB1	0.008866258
1230	RUFY3	0.008845214
1231	C9orf30	0.008842197

1232	YWHAZ	0.008822886
1233	ZNF250	0.008812364
1234	RAB2A	0.008793056
1235	NUDT1	0.008779469
1236	ARRDC3	0.00877423
1237	VAMP2	0.008715205
1238	MGC18216	0.008698422
1239	RFNG	0.008618612
1240	TROAP	0.008617588
1241	AP1G2	0.008615644
1242	PTTG1	0.008596581
1243	TBL2	0.008571566
1244	PLEKHO1	0.008556368
1245	SUMF1	0.00854998
1246	C10orf119	0.008546816
1247	PPP3CA	0.008539166
1248	IDH3B	0.008533645
1249	MBOAT2	0.008522718
1250	DCTN6	0.008502529
1251	KMO	0.008496771
1252	FBXO30	0.008494517
1253	KRAS	0.008447561
1254	CLSTN1	0.008419709
1255	PAPD1	0.008377329
1256	HIST1H4D	0.008364075
1257	EXOSC6	0.008324399
1258	MR1	0.008310639
1259	CCM2	0.008297909
1260	CD4	0.008272281
1261	L3MBTL2	0.008263224
1262	TNFSF10	0.008246884
1263	LOC653381	0.008204727
1264	FGF9	0.008195646
1265	RNPS1	0.008182027
1266	DMWD	0.008158235
1267	C18orf25	0.008147028
1268	LRSAM1	0.00813731
1269	TBC1D14	0.008109965
1270	TRERF1	0.00810631
1271	ISY1	0.008086188
1272	LOC653489	0.008063727
1273	PLXNB1	0.008042927
1274	ZNF655	0.008028424
1275	IMPDH1	0.008012719

1276	MRPS18C	0.00800896
1277	KIAA0753	0.007997148
1278	ZNF766	0.007986621
1279	ACOT9	0.007970508
1280	MAT2B	0.007968793
1281	CHMP4B	0.007934983
1282	C1orf108	0.007926851
1283	PARP11	0.007865517
1284	NDUFS7	0.00784798
1285	RAB31	0.007798489
1286	TXNRD1	0.007788276
1287	PPP2R2B	0.007783691
1288	LARP1	0.00776552
1289	PTPN12	0.007756417
1290	PKN1	0.007745196
1291	TLR8	0.007711188
1292	C8orf37	0.007706942
1293	POLQ	0.007704428
1294	KIF1B	0.007700739
1295	PLEKHF1	0.007684927
1296	PSMF1	0.007657777
1297	NCF4	0.007646165
1298	GPR109B	0.007629804
1299	PNPO	0.007607095
1300	CMIP	0.007597155
1301	LOC652489	0.007594061
1302	DICER1	0.00757869
1303	PBXIP1	0.007552673
1304	C11orf48	0.007531799
1305	FAM20C	0.007514838
1306	ARHGAP25	0.007497664
1307	RXRG	0.007479575
1308	GTDC1	0.0074719
1309	SULT1A1	0.007467143
1310	CBX3	0.007436579
1311	SFI1	0.007428917
1312	GBE1	0.007355103
1313	GPR1	0.00728505
1314	PAPD4	0.007272772
1315	ZNF275	0.007259716
1316	C4orf32	0.007222125
1317	GRK5	0.007215675
1318	CENTD2	0.007142817
1319	ERO1L	0.007131711

1320	KIAA1751	0.007114423
1321	KEAP1	0.00706955
1322	SNX11	0.007068586
1323	PANK2	0.007062918
1324	ERCC1	0.007051428
1325	BUB1	0.007030262
1326	NOL1	0.007014556
1327	OSBPL7	0.007005363
1328	UBE3B	0.006995409
1329	VPS26A	0.006960535
1330	DUSP8	0.006947716
1331	RAD23B	0.00694376
1332	CR1	0.006920168
1333	SEC24A	0.006915167
1334	LOC286208	0.006907798
1335	Septin.2	0.006898051
1336	DHRS4	0.006861588
1337	LOC654346	0.006843237
1338	LOC650739	0.006817977
1339	RAP2C	0.006816534
1340	CALML4	0.006799767
1341	SMOC1	0.006797301
1342	CACYBP	0.006791075
1343	PFKFB3	0.006755015
1344	CIP29	0.00671978
1345	C10orf76	0.006672749
1346	ELMO1	0.006624717
1347	ENTPD6	0.006624408
1348	TOR1AIP1	0.006609555
1349	LOC648249	0.006564074
1350	HTR2A	0.006558628
1351	MZF1	0.00653798
1352	C19orf48	0.006507433
1353	SCFD1	0.006495435
1354	MSRA	0.006477263
1355	INTS8	0.006461705
1356	TNRC6B	0.006411369
1357	NGRN	0.006407587
1358	DCLRE1C	0.006379104
1359	C18orf24	0.006356631
1360	RABL2B	0.006349864
1361	FIP1L1	0.006335019
1362	DNASE1L1	0.006321153
1363	TRADD	0.006312053

1364	SLC25A25	0.006291953
1365	BANF1	0.006290446
1366	DIAPH2	0.006278479
1367	ANXA7	0.006262228
1368	VEGFB	0.006257115
1369	CD38	0.006196103
1370	NUS1	0.006176098
1371	OTUB1	0.006175526
1372	JARID1A	0.006169679
1373	ATP11B	0.006162326
1374	LOC647349	0.006151536
1375	ILF3	0.006130852
1376	ATP1A1	0.006104243
1377	COMT	0.006095764
1378	PHF23	0.006087174
1379	C6orf106	0.006063687
1380	THAP7	0.006056223
1381	DNAJC5	0.006046054
1382	CCNB1IP1	0.006044701
1383	AGPAT1	0.006025722
1384	CD58	0.006009982
1385	LILRB1	0.005970483
1386	AMY1A	0.00595138
1387	APOL3	0.00592083
1388	HDGF2	0.00591703
1389	ERN1	0.005865675
1390	FAM115A	0.005844747
1391	NBPF20	0.005839791
1392	FLAD1	0.005829248
1393	ANKMY1	0.005826008
1394	CYB5R4	0.005774265
1395	KCTD18	0.0057705
1396	SNHG10	0.005770351
1397	GNPTAB	0.005754393
1398	UBN1	0.005668834
1399	CTNS	0.005633919
1400	EIF2B4	0.005627726
1401	XRN2	0.005610916
1402	MCM7	0.005582143
1403	BMS1	0.005544674
1404	APPL1	0.005519975
1405	PPP2R5C	0.005483373
1406	TRAF3IP2	0.005381979
1407	EAF1	0.005341584

1408	LOC647856	0.005292656
1409	PTP4A2	0.005286907
1410	MCM8	0.005259822
1411	DBNDD2	0.005213247
1412	ZMYND11	0.005182114
1413	SLC2A14	0.005155345
1414	PREPL	0.005146117
1415	PEMT	0.005131908
1416	FLI1	0.005105292
1417	PHF13	0.005035184
1418	LOC387856	0.005030211
1419	UBE2G2	0.005008785
1420	DR1	0.004973283
1421	COQ6	0.004926606
1422	FKSG30	0.004895988
1423	TADA3L	0.004873728
1424	ALDOA	0.004825061
1425	C21orf59	0.004819018
1426	ARPC4	0.00480946
1427	VARS	0.004745816
1428	OPRL1	0.004730539
1429	DDX19B	0.004717386
1430	ST3GAL1	0.004698379
1431	NGLY1	0.004697001
1432	DHTKD1	0.004645243
1433	LOC644935	0.004606404
1434	ARHGEF3	0.00460185
1435	NLRP8	0.004598584
1436	UBE2E3	0.004482603
1437	NIPBL	0.004482271
1438	SHOC2	0.004440879
1439	FKBP2	0.0043998
1440	UBE2Z	0.004391192
1441	RAPGEF1	0.00435577
1442	SPN	0.004323962
1443	SCAMP3	0.004252011
1444	HM13	0.004219953
1445	RPS6KB2	0.004196337
1446	MRPS12	0.004190696
1447	CUTA	0.004185246
1448	FAM102B	0.004180684
1449	C14orf173	0.004115001
1450	UBQLN2	0.004103036
1451	C1orf2	0.004099777

1452	TRAF7	0.004038803
1453	ATP1B3	0.004033302
1454	LAMA1	0.003998386
1455	SMARCD1	0.003939291
1456	LCOR	0.003892629
1457	DIABLO	0.003878578
1458	PPM1D	0.003866138
1459	CPSF4	0.003846617
1460	SGK3	0.003697244
1461	TOX4	0.003690326
1462	DHX30	0.00363938
1463	STARD7	0.003615263
1464	C3orf37	0.003609495
1465	TMEM199	0.003577232
1466	DDX39	0.003547998
1467	EBAG9	0.003517708
1468	BTBD11	0.003505194
1469	NAT5	0.003491131
1470	SDF4	0.00348142
1471	DPH2	0.003356384
1472	MAGED1	0.003275892
1473	MTPN	0.003265035
1474	COMMD4	0.003242396
1475	STAR	0.003237361
1476	TBCD	0.003229147
1477	RAB8B	0.003127791
1478	RPS26L1	0.003124002
1479	KLHL24	0.003021265
1480	SCYL2	0.002980335
1481	MAP3K7IP1	0.002916735
1482	PTPN7	0.002899387
1483	ASPSCR1	0.00288112
1484	DDX42	0.002817087
1485	UCHL5IP	0.002530182
